# Spongostan^™^ Leads to Increased Regeneration of a Rat Calvarial Critical Size Defect Compared to NanoBone^®^ and Actifuse

**DOI:** 10.3390/ma14081961

**Published:** 2021-04-14

**Authors:** Dirk Wähnert, Julian Koettnitz, Madlen Merten, Daniel Kronenberg, Richard Stange, Johannes F. W. Greiner, Christian Kaltschmidt, Thomas Vordemvenne, Barbara Kaltschmidt

**Affiliations:** 1 Protestant Hospital of Bethel Foundation, Department of Trauma and Orthopedic Surgery, University Hospital OWL of Bielefeld University, Campus Bielefeld-Bethel, Burgsteig 13, 33617 Bielefeld, Germany; Dirk.Waehnert@evkb.de (D.W.); Julian.Koettnitz@evkb.de (J.K.); Thomas.Vordemvenne@evkb.de (T.V.); 2 Molecular Neurobiology, Bielefeld University, Universitätsstrasse 25, 33615 Bielefeld, Germany; Madlen.Merten@uni-bielefeld.de; 3 Department of Regenerative Musculoskeletal Medicine, Institute for Musculoskeletal Medicine, University Hospital Muenster, Westfaelische Wilhelms University Muenster, Albert-Schweitzer-Campus 1, Building D3, 48149 Muenster, Germany; Daniel.Kronenberg@ukmuenster.de (D.K.); Richard.Stange@ukmuenster.de (R.S.); 4 Department of Cell Biology, Bielefeld University, Universitätsstrasse 25, 33615 Bielefeld, Germany; Johannes.Greiner@uni-bielefeld.de (J.F.W.G.); C.Kaltschmidt@uni-bielefeld.de (C.K.)

**Keywords:** bone regeneration, critical size defect, rat calvaria, bone substitute materials

## Abstract

Bone substitute materials are becoming increasingly important in oral and maxillofacial surgery. Reconstruction of critical size bone defects is still challenging for surgeons. Here, we compared the clinically applied organic bone substitute materials NanoBone^®^ (nanocrystalline hydroxyapatite and nanostructured silica gel; *n* = 5) and Actifuse (calcium phosphate with silicate substitution; *n* = 5) with natural collagen-based Spongostan™ (hardened pork gelatin containing formalin and lauryl alcohol; *n* = 5) in bilateral rat critical-size defects (5 mm diameter). On topological level, NanoBone is known to harbour nanopores of about 20 nm diameter, while Actifuse comprises micropores of 200–500 µm. Spongostan™, which is clinically applied as a haemostatic agent, combines in its wet form both nano- and microporous topological features by comprising 60.66 ± 24.48 μm micropores accompanied by nanopores of 32.97 ± 1.41 nm diameter. Micro-computed tomography (µCT) used for evaluation 30 days after surgery revealed a significant increase in bone volume by all three bone substitute materials in comparison to the untreated controls. Clearly visual was the closure of trepanation in all treated groups, but granular appearance of NanoBone^®^ and Actifuse with less closure at the margins of the burr holes. In contrast, transplantion of Spongostan™ lead to complete filling of the burr hole with the highest bone volume of 7.98 ccm and the highest bone mineral density compared to all other groups. In summary, transplantation of Spongostan™ resulted in increased regeneration of a rat calvarial critical size defect compared to NanoBone and Actifuse, suggesting the distinct nano- and microtopography of wet Spongostan™ to account for this superior regenerative capacity. Since Spongostan™ is a clinically approved product used primarily for haemostasis, it may represent an interesting alternative in the reconstruction of defects in the maxillary region.

## 1. Introduction

Bone healing and implant integration are highly regulated processes that are based on optimal conditions, both biological and mechanical. Impaired bone healing and implant integration are still a major challenge in dental surgery [1,2,3]. Therefore, worldwide bone grafting procedures represent the second most frequent tissue transplantation (right after blood transfusion) with over two million procedures performed every year, causing estimated cost of $2.5 billion [4,5].

In bony defect repair, autologous bone grafting is still the “gold standard” since it combines all requirements for ideal bone substitute materials: biocompatibility, bioresorbabilty, osteoconductivity, osteoinductivity as well as structural and mechanical similarity to bone [6]. In dental surgery problems arise when alveolar ridge augmentation is needed due to the limited bone volume in the jaw. Especially in this context guided bone regeneration is the most used technique. But current bone substitutes for implant dentistry provide only osteoconduction but not osteoinduction [7]. A promising further strategy might involve the use of mesenchymal stem cells (MCS) [8].

Many bone substitute materials have been developed that differ decidedly in composition and structure. These materials can be divided into five groups: materials of natural origin, synthetic materials, composite materials, materials combined with growth factors and materials loaded with living cells. All these materials are used in clinical practice with different indications and variable successes [6,9,10,11].

Despite the broad range of available substitutes, engineering bone substitute materials is a complex process, since their nano- and microtopography need to allow optimal vascular ingrowth as well as stem cell and osteogenic progenitor cell migration and differentiation. Recently, we demonstrated the capability of 31.93 ± 0.97 nm pores on the surface of rat collagen type I fibres to induce osteogenic differentiation of adult human stem cells in vitro [12]. Petersen and colleagues reported significantly improved endochondral ossification in a critical-size defect model at the rat femur for an engineered porcine collagen scaffold with a channel-like pore architecture comprising micropores of 89 ± 15 μm [13]. Taking advantage of this knowledge, we very recently identified the optimal nano- and microtopography guiding intramembranous ossification-driven bone regeneration. In particular, we observed micropores of 60.66 ± 24.48 μm diameter and nanopores of 32.97 ± 1.41 nm size in the clinically approved collagen sponge Spongostan™ [14], which is commonly used as a haemostatic collagen sponge in a broad range of clinical settings [15,16,17]. A specific indication for the use of Spongostan as a haemostatic agent is for bleeding in patients with von Willebrand disease (vWD). A site of frequent bleeding in these patients is within the nose. Treatment of patients with vWD is challenging; due to frequent mucosal bleeding, but dental procedures are often required, and the use of Spongostan is indicated. Treatment of these patients should be adjusted to prevent bleeding and its complications as much as possible (replacement therapy and haemostatic agents [18]. 

Beyond the applicability of Spongostan™ as a stem cell or growth factor carrier for bone recovery [19,20], we demonstrated the sole presence of micro- and nanotopographical cues on Spongostan™ to be sufficient for functional regeneration of a critical-size calvarial rat bone defect [14]. 

The 5 mm biparietal defect in the rat skull can be considered as a critical-size defect because, as shown in a systematic review by Vajgel et al., it has a very low rate (1.6%) of spontaneous complete healing [21]. Thus, the critical-size rat calvarial defect is a well-established and accepted animal model for the in vivo study of bone regeneration in dental surgery as well [22,23,24,25].

Since, all available material for bone regeneration in dental surgery have shown to be only osteoconductive [7] we tested the regenerative capacity of Spongostan™ harbouring both micro- and nanopores with the two clinically proven bone substitute materials NanoBone^®^ and Actifuse in a rat calvarial critical size defect model. The synthetic NanoBone^®^, a nanocrystalline hydroxylapatite (HA) similar to the autologous HA embedded in a highly porous silica gel matrix, is routinely used for guiding intramembranous ossification during bone reconstruction in craniofacial surgery [26,27,28]. NanoBone^®^ is considered to be biocompatible, non-immunogenic, osteoconductive and osteoinductive [27]. In the nanoscale, the silica gel matrix of NanoBone^®^ has interconnecting pores with sizes ranging from 10 to 20 nm, resulting in a great porosity of surface [29]. Next to NanoBone^®^, Actifuse, a porous version of Si-HA harbouring micropores of 200–500 µm diameter, also fulfils the requirements for bone substitute materials introduced above. Being broadly applied as a bone graft substitute for application in spinal fusion operations [30,31,32] its particularly slow degradation rate makes Actifuse an excellent bone augmentation material for endochondral ossification [31,33]. 

The aim of this work was to evaluate the new bone volume formed in vivo by clinically established (osteoconductive) bone graft substitutes with Spongostan™, which has an additional osteoinductive effect due to its micro- and nanotopograpy.

## 2. Materials and Methods

Parts of the results for the Spongostan™ and control group have been published in the study of Vordemvenne and co-authors [14]. 

### 2.1. Bone Substitute Materials 

NanoBone^®^ (NanoBone^®^ granulate, ARTOSS GmbH, Rostock, Germany) is a synthetic bone substitute with two components: 76% nanocrystalline hydroxyapatite and 24% nanostructured silica gel comprising nanopores of 10–20 nm diameter. For this study the fine granule with a diameter of 0.6 mm were used.

Actifuse (Actifuse ABX, Baxter Germany, Unterschleißheim, Germany) is a synthetic calcium phosphate bone substitute with silicate substitution. The material has a granule size of 1–2 mm and comprises micropores of 200–500 µm.

Spongostan™ (Ferrosan Medical Devices, Søborg, Denmark; marketed by Ethicon Biosurgery, Johnson and Johnson, New Brunswick, NJ, USA) is a compound of hardened pork gelatin containing formalin (1.5%) and lauryl alcohol (2%). For this investigation, pre-wetted Spongostan™ cubes of 1 mm^3^ containing micropores of 60.66 ± 24.48 μm diameter and nanopores of 32.97 ± 1.41 nm have been used as described by Vordemvenne et al. [14].

### 2.2. Animals

The responsible authority (LANUV, Approval Ref. No. 81-02.04.2018.A188) approved this study. All animal work followed the policies and procedures established by the Animal Welfare act, the National Institutes of Health Guide for Care and Use of Laboratory Animals, and the National Animal Welfare Guidelines.

Total of 20 male white Wistar rats with an average weight of 300 g at the day of surgery and an age of about 8 weeks were used for this study. The sample size was calculated using G*power software (version 3.1.9.2, 2018, Heinrich Heine University, Düsseldorf, Germany, 1 May 2018) (one-way ANOVA test with a priori analysis: α = 0.05, power = 0.8, effect size f = 0.75) under the supervision of a statistician. The animals were assigned to either group A (empty control, *n* = 5), group B (NanoBone^®^, *n* = 5), group C (Actifuse, *n* = 5), or group D (Spongostan™, *n* = 5) by randomisation. The animals are from our own breeding at the University of Bielefeld. They were housed with food and water provided ad libitum, under a 12 h light/dark cycle with two animals per cage. Following the 3R (replacement, reduction, refinement) guidelines all efforts were made to minimise potential suffering and a minimum number of animals was used for this study.

### 2.3. Animal Surgery

The surgical procedures followed our previously described protocol [14] and was modified according to the description of Spicer et al. [23]. The critical size calvarial defect is an established model for the investigation of bone regeneration in dental surgery [22,23].

**Figure 1:**
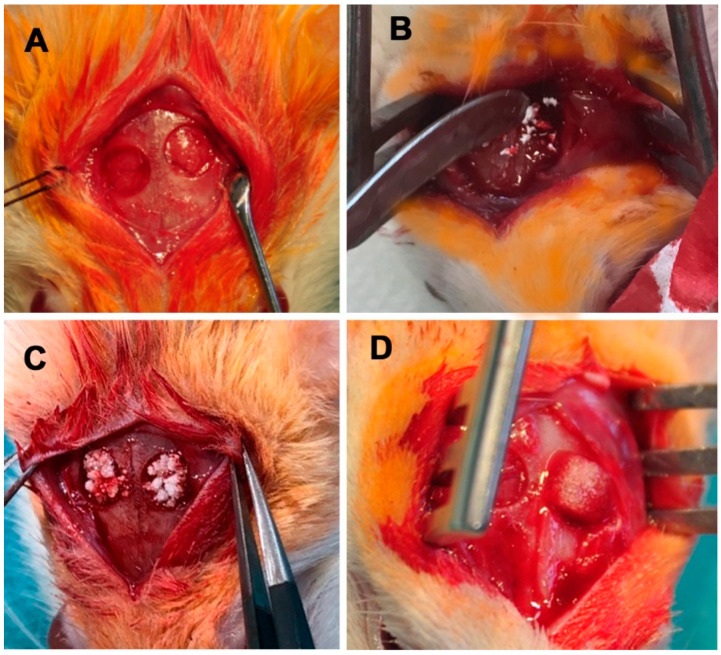
Photograph showing the bilateral critical size-defect with (**A**) empty holes (control group), (**B**) NanoBone^®^, (**C**) Actifuse, and (**D**) pre-soaked Spongostan™.

Anaesthesia was performed using an intraperitoneal injection of ketamine hydrochloride (60 mg/kg, Inresa Arzneimittel GmbH, Freiburg, Germany) and medetomidine (0.3 mg/kg, Dechra Veterinary Products Ltd., Shrewsbury, UK) after subcutaneous injection of tramadol (20 mg/kg, Grünenthal GmbH, Aachen, Germany). For preparing the area of surgery, an electric clipper was used to shave the area of surgery. Afterwards, the animal was placed on the operating table on a sterile covered heat plate (37 °C). After cleaning the area of surgery using an iodine swab, eyes were protected by using eye ointment (Bepanthen Augensalbe, Bayer AG, Berlin, Germany). A 2 cm skin incision was made in the midline of the skull (from the occipital bone to the base of the nose). After preparation of the subcutaneous tissue, the periosteum was split at the sagittal midline using a scalpel. After mobilisation of the periosteum, holding sutures (Vicryl Plus 3-0, Ethicon, Johnson and Johnson, New Brunswick, NJ, USA) were positioned through it allowing lateral traction and protecting skin and periosteum. Trepanation was performed bilaterally using a 5 mm hollow drill (Trephines 229 RAL 040: Hager and Meisinger GmbH, Neuss, Germany; Implantmed: W&H Dentalwerk Bürmoos GmbH, Bürmoos, Austria) with a constant speed of 2000 rpm. Sterile saline solution was applied dropwise to the bone to avoid heating. Trepanation using the drill was performed until the near full thickness was reached, it was completed using the elevator to lift out the bone flap (Figure 1). After washing the defect with sterile saline solution, the holes were treated according to the group assigned to the animal (empty, NanoBone^®^, Actifuse, or Spongostan™). Wound closure was performed layer by layer, the closure of the periosteum was performed using running sutures (Prolene 5-0, Ethicon, Johnson and Johnson, New Brunswick, NJ, USA). Skin closure was performed by single back-and-forth sutures (Prolene 3-0, Ethicon, Johnson and Johnson, New Brunswick, NJ, USA). Finally, the area of surgery was cleaned using sterile saline solution.

### 2.4. Postoperative Care 

Postoperatively, rats have been placed in a prewarmed cage. All animals were housed alone for 3 days. For 5 days postoperatively, animals received analgetic drugs in their water bottles (Tramadol, Grünenthal GmbH, Aachen, Germany, 2.5 mg/100 mL drinking water, sweetened with 5% sugar). 

### 2.5. Euthanasia and Sample Extraction

Euthanasia was performed 30 days postoperatively using the Exposure Line carbon dioxide box bioscape (Ehret, Freiburg, Germany). The calvarium was dissected by removing the soft tissue and the surrounding bone. Specimens were stored in phosphate buffered saline (PBS) until micro-CT was performed. Afterwards specimens were fixated and stored using 4% paraformaldehyde (PFA).

### 2.6. Micro-CT

**Figure 2:**
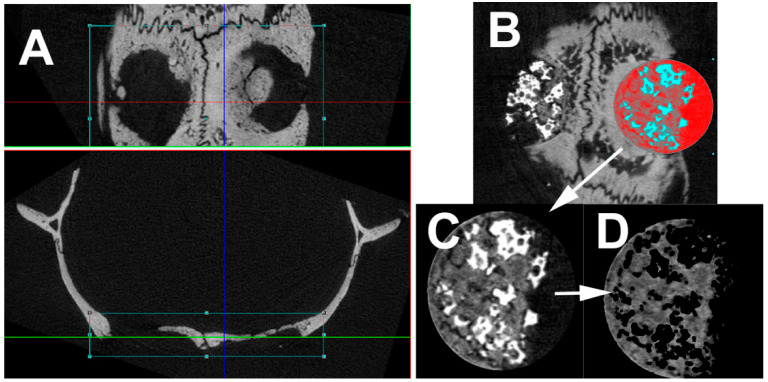
Picture showing the micro-computed tomography (CT) evaluation. (**A**) First the slices have been placed in correct orientation for reconstruction. (**B**) Thereafter a circular region of interest (diameter 5 mm) has been placed into the defect. (**C**) by defining the first and last slice with bone, the volume of interest was defined. (**D**) Semi-automated evaluation protocol removed very dense regions (Actifuse, NanoBone^®^) as well as air and evaluated the volume of interest with respect to the defined parameters.

After extraction, a micro-computed tomography (μCT) was performed of all specimens (SkySkan 1176, Bruker, Kontich, Belgium). The following parameters have been used: isotropic resolution of 8.9 μm, 50 kV energy at 500 μA intensity. An angle shift of 0.5° per image was used. To reduce artefacts an average of five pictures per angle was used. The alignment of the specimens was standardised, allowing transversal slices to be acquired. Axial reconstruction was applied to the images for further evaluation. The volume and density of the new bone formation was evaluated in a volume of interest (VOI). To define this, first, into each defect a circular region of interest (ROI) was placed (Figure 2). Afterwards, the VOI was set by the first and last slice in the Z-direction with the appearance of bone. Newly formed bone volume, and bone mineral density (BMD) have been quantified by a standardised semi-automated protocol for each VOI (Figure 2). The mean values of the right and left hole was used for further evaluation. For standardised BMD evaluation, the µCT was calibrated with a two-density phantom (0.25 g hydroxyapatite/ccm and 0.75 g hydroxyapatite/ccm). Additionally, the intact BMD was evaluated within two VOIs, one anterior and one posterior, to each defect.

### 2.7. Statistics

GraphPad Prism software (Prism version 3.0.1, 2020, GraphPad, San Diego, CA, USA) was applied for statistical evaluation of measured bone volumes and bone mineral density in individual defects. The Shapiro-Wilk test was applied to test the data for normal distribution. Normal distribution had to be rejected, so the Mann-Whitney test was used to test for significant differences in the groups in terms of newly formed bone volume and bone mineral density. Here, ** *p* < 0.05 was considered significant. 

## 3. Results

Critical size calvarial defects show increased regeneration upon transplantation of Spongostan™ compared to NanoBone^®^ and Actifuse.

**Figure 3:**
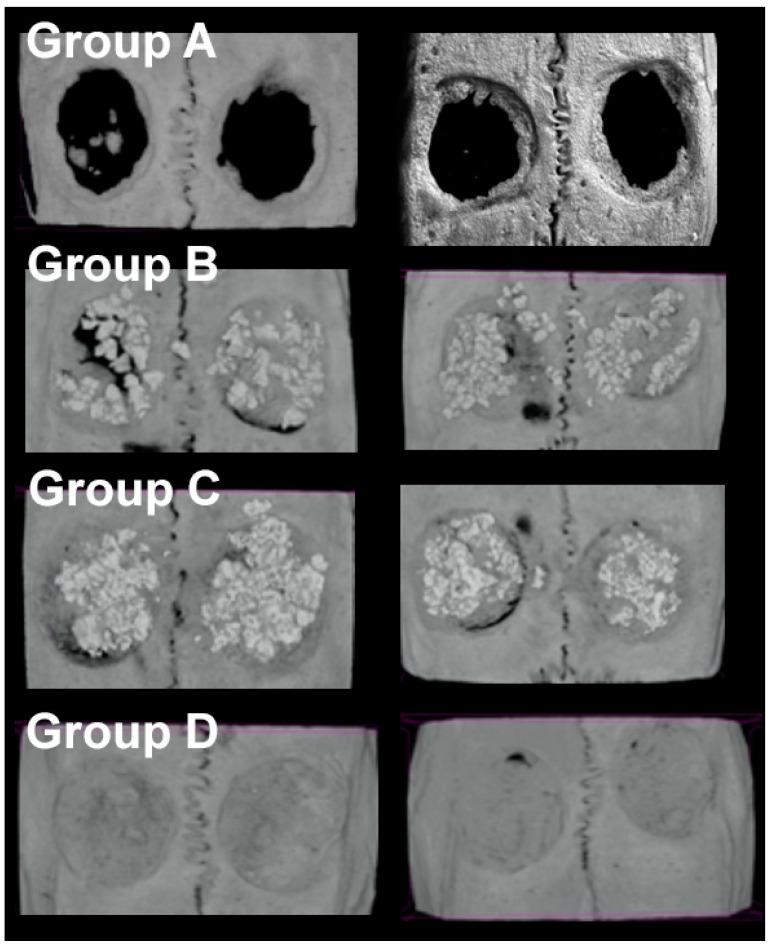
3D reconstructions of the micro-CT showing the results of two examples of each group. Transplantation of Spongostan™ results in significantly increased mineral density of the new bone compared to NanoBone^®^ and Actifuse. Group A—the control group; Group B—NanoBone^®^; Group C—Actifuse; Group D—Spongostan™.

**Figure 4:**
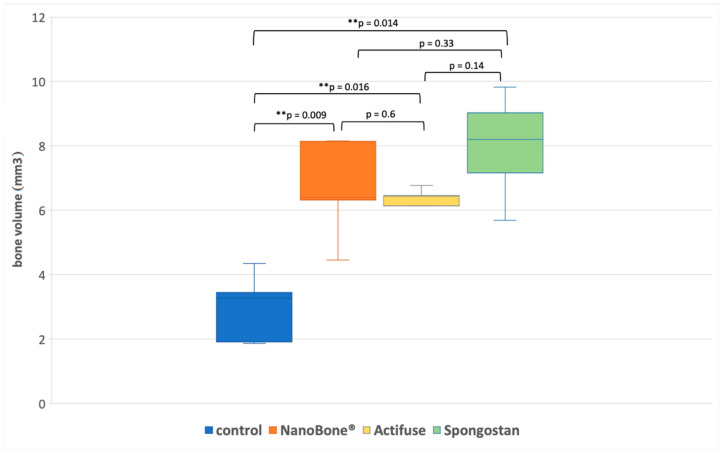
Box-plot diagram of the newly formed bone volume with respect to the treatment group.

The newly formed bone volume differed significantly between the groups (Table 1, Figure 3 and Figure 4). The control group (group A) showed the lowest bone volume compared to all other groups (*p* ≤ 0.016). Group B (NanoBone^®^) and C (Actifuse) showed significantly more newly formed bone volume compared to group A (control) with 125% (*p* = 0.009) and 103% (*p* = 0.016) increase, respectively. The highest bone volume was found in group D (Spongostan™) with 169% increase which was significantly elevated compared to group A (control, *p* = 0.014). 

**Figure 5:**
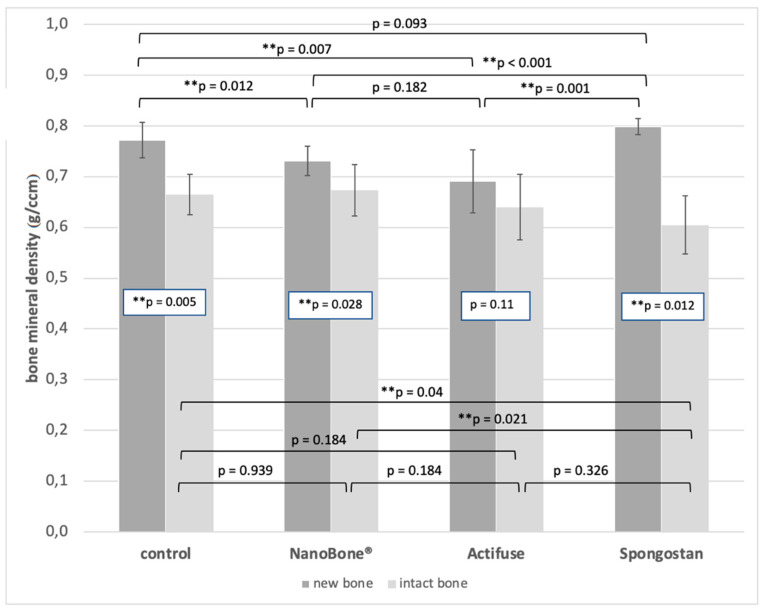
Diagram showing the bone mineral density of the new bone and the intact bone with respect to the treatment groups. *p*-values for the comparison new vs. intact bone are shown within the bars. Significant differences between the groups are marked (*) above the bars.

Comparing the bone mineral density (BMD) of the newly formed and the intact bone, significant differences with higher BMD values for the newly formed bone were found for group A (control) (*p* = 0.005), group B (NanoBone^®^) (*p* = 0.028), and group D (Spongostan™) (*p* = 0.012, Figure 5, Table 2).

Comparing the BMD of the newly formed bone between the groups (Figure 5, Table 2), significant higher values were found for group D (Spongostan™) compared to groups B (NanoBone^®^, *p* = 0.001), C (Actifuse, *p* = 0.001), as well as for group A (control) compared to group B (NanoBone^®^, *p* = 0.012) and group C (Actifuse, *p* = 0.007).

Comparing the BMD of the intact bone between the groups (Figure 5, Table 2), the significant lowest value was found for the Spongostan group compared to the control (*p* = 0.04) and NanoBone^®^ (*p* = 0.021) group. All other differences were not statistically significant.

Evaluation of histological sections stained with trichrome staining according to Goldner, with nuclei were stained with hematein did not reveal formation of inflammatory infiltrates (data not shown). In addition we performed staining with antibodies against the proinflammatory transcription factor NF-kB RelA, here also no reactive nuclei were detected (data not shown). Taken together, 30 days after surgery no signs of inflammation were detected at the side of surgery.

## 4. Discussion

The regeneration of dental bone defects remains a clinical challenge [34]. The demanding anatomical conditions of the jaw present surgeons with great challenges in the reconstruction of bone defects [35]. Large or critical size defects are defined as defects, where the physiological regenerative capability is exceeded and therefore, healing will not occur without intervention [21,36]. The cause of such defects can be trauma, tumour or congenital malformations [37,38,39,40]. These are so common that bony defects are part of everyday oral and maxillofacial surgery [35,41]. Sufficient reconstruction of the defect situation is essential, as inadequate or untreated defects have a major impact on the function, aesthetics, quality of life, and psychology of the patient.

Since the currently available bone graft substitutes (e.g., nanostructured calcium apatite-NanoBone, Actifuse) do not possess osteoinductive properties, we compared the innovative bone substitute material Spongostan™ with clinically established materials in a critical size-defect model at the calvaria of the rat. While the clinically proven bone substitute materials NanoBone^®^ and Actifuse increased the new bone volume by 125% and 103%, transplantation of Spongostan™ even resulted in bone volume increase of 169% compared to control. In addition, we observed a significantly increased bone mineral density of the new bone by Spongostan™ compared to NanoBone^®^ and Actifuse after transplantation into calvarial critical size defects. The control group showed significantly higher bone mineral density of the newly formed bone compared to the NanoBone^®^ and Actifuse group. This may be the effect of relatively small volume of newly formed bone in control group, leading to overestimation of bone density. Therefore, there is also no significant difference with the Spongostan™ group. Regarding the bone mineral density of the intact bone surrounding the defect, we found significant lower values in the Spongostan group compared to the control and NanoBone^®^ group. This effect may result from the mobilization of hydroxyapatite from the surrounding bone tissue for calcification of the defect in the Spongostan™ group. Spongostan™, unlike Actifuse and NanoBone^®^, does not release components for calcification of the extracellular matrix during resorption/degradation.

Bone substitute materials for routine clinical use can be divided according to their origin as follows: materials of natural origin (autogen, allogen, xenogen, phytogenic), synthetic materials (calcium phosphate, calcium sulphate, bioglass, polymers) and composite materials. To all of them the requirements are the same: Ideal bone substitute materials have to be biocompatible, osteoconductive, osteoinductive, resorbable and mechanically stable. With the so called ‘diamond concept’ the group of Giannoudis et al. introduced a framework with essentials for successful bone healing in orthopaedics. This concept includes vascularity and host factors, both have significant impact on the healing potential [42,43,44,45]. Additionally, osteoinductive mediators, osteogenic cells, osteoconductive matrix and mechanical stability are essential factors within this concept. All together forming the ‘diamond concept’, which has shown to be particularly useful in the management of non-unions at the upper and lower extremities [46,47,48,49]. We suggest that some aspects of the diamond concept are also for the concerns in the reconstruction of bone defects on the jaw.

Ceramic bone substitutes (such as NanoBone^®^ and Actifuse) consist of inorganic, non-metallic materials. Since 70% of bone tissue consists of hydroxyapatite, these calcium phosphate ceramics obtain their bioactivity by mimicking these mineral bone components [50]. They provide a suitable surface for new bone formation. The mechanical properties of ceramic bone substitutes are characterised by a higher mechanical strength than human cortical bone. However, they show lower toughness and higher modulus of elasticity than cortical bone [51]. Calcium phosphate ceramics are usually made of hydroxyapatite, tricalcium phosphate or a combination. The composition is similar to that of natural bone tissue, which explains the good biocompatibility and osteoconductivity [52]. Ceramic bone substitutes can be well osteointegrated. The degradation or resorption of ceramic bone substitutes is strongly dependent on the composition. Hydroxyapatite is characterised by high crystallinity, which makes it difficult to degrade in vivo. In contrast, tricalcium phosphate consists of (alpha and beta) crystals with different crystallography and resorption behaviour. Overall, tricalcium phosphate is more readily degradable than hydroxyapatite and becomes soluble more quickly [53]. The two ceramic bone substitute materials investigated have silicate as an additional component. It is known that silicate is an essential component for the mineralisation of bone [54]. Furthermore, it was shown in vivo that the addition of zinc silicate to a collagen-hydroxyapatite bone graft substitute created a favourable osteogenic microenvironment and enhanced bone angiogenesis [55].

Collagen, the main component of Spongostan™, is one of the natural polymeric bone substitutes. Other natural polymers include chitosan, alginate, elastin and cellulose [50]. These bone substitute materials can be completely replaced by new bone tissue [56]. Resorbability is based on enzymatic or hydrolytic degradation [57]. Collagen is the most widely used bone substitute material of this group. Collagen is the main component of the extracellular matrix and is therefore non-toxic. It is also easy to isolate and has high biocompatibility and low immunogenicity [58]. The mechanical properties of collagen are poor, and its hydrophilicity can lead to swelling [50].

Each bone graft substitute has its advantages and disadvantages. Ceramics are characterised by their good mechanical properties, but they are brittle and have low plasticity and show insufficient degradation. Polymers, on the other hand, are very well biocompatible and show excellent biodegradation, but their mechanical properties are highly variable and depend on the material chosen as well as the production. All synthetic bone substitute materials have one major disadvantage, inadequate vascularization and consequent poor nutrient transport to the interior of the material.

Especially, in term of its osteoinductive capacity, the nano- and microtopography of a bone substitute material might be of great interest in order to mimic the unique nano- and microtopography of natural bone as closely as possible. In this regard, changes in the nanotopography of artificial surfaces were reported to directly regulate differentiation of stem cells [59,60,61,62,63,64]. Accordingly, we were able to show the potential of a defined nanotopography of about 30 nm pores present on the surface of collagen to induce osteogenic differentiation of human inferior turbinate as well as human mesenchymal stem cells in our previous work [12,14]. This property can be called osteogenicity or osteopromotive [4]. Interestingly, the here applied bone substitute material NanoBone^®^ is likewise known to have a porous surface in the nanoscale, although pore diameters (10–20 nm, [29]) are smaller than those present on Spongostan™ (30 nm, [14]). However, the presence of interconnecting nanopores between SiO_2_-connected HA crystals was likewise suggested to provide mechanic stability, bioresorption and osteoconduction of NanoBone^®^ [29]. Nevertheless, we suggest the here-observed increase in bone recovery after transplantation of Spongostan™ compared to NanoBone^®^ to be based at least in part on these differences in surface nanotopography. 

Next to the nanoscale, the microtopography is likewise crucial for successful bony healing. It is well-known that pores with a diameter of at least 100 µm allow vascular ingrowth [65,66]. In addition, micropores of a bone substitute material treatment are recommended to range between 200 and 500 μm, to allow osteointegration [67,68,69]. In this line, Actifuse is known to harbour micropores ranging between 200 and 500 µm [70]. Our previous results further indicate even smaller micropores of 60.66 ± 24.48 μm diameter to be advantageous for cell adhesion and incorporation [14]. In accordance to our observations, Petersen et al. demonstrated a microporous scaffold from porcine collagen with channel like pores with a size of 89 ± 15 µm to efficiently induce endochondral ossification in a critical size-defect model at the rat femur, although a complete bone regeneration was not achieved [13]. Overcoming this challenge, the here applied collagen sponge Spongostan™ combines both nano- and microtopological features and resulted in a complete regeneration of calvarial critical size-defect after 30 days [14]. Thus, application of Spongostan™ also resulted in the best calvarial bone regeneration compared to NanoBone^®^ and Actifuse in the present study. We therefore likewise suggest the combination of the distinct nano- and microtopography of Spongostan™ to account for the observed increase in bone recovery. 

This study also has limitations, one being a small number of animals and the other being a singular evaluation point. Despite the small number of animals (*n* = 5 per group), we were able to show significant differences between the groups, so that a larger number would not have provided any additional information. In the clinical context, rapid and sufficient bone formation is important, so in the present study the newly formed bone volume was evaluated after 30 days, knowing that the resorption and degradation properties of the investigated bone graft substitutes differ significantly. This approach is also well-known in the literature, since 6 out of 22 studies using the 5 mm critical-size bone defect in the rat calvaria have chosen a singular (1 month) evaluation time point only [21].

Another factor in studies of critical size bone defects is generation. All measures must be taken to avoid thermally induced bone necrosis. Therefore, all trepanations in this study have been performed by two experienced surgeons. To avoid thermal necrosis we choose a speed of 2000 rpm in combination with constant saline irrigation (as most important factor to avoid thermal necrosis as shown by Augustin et al. [71]) and the so called impact drilling technology. The drill is repeatedly lifted to allow the rinsing solution to penetrate under the drill and thus ensure sufficient cooling. In addition, the bone density and thickness as well as the force applied to the drill are decisive parameters for the temperature development. Since the skull thickness of the rat is less then 1 mm and therefore the force exerted on the drill is low, the temperature development is also reduced. Chen et al. even reported lower temperatures when using higher drilling speeds due to faster drilling and thus lower friction contact time [72]. Taking all these parameters together, we believe that we have largely minimised the risk of thermal osteonecrosis with the drilling technique we have used.

Off note, bridging critical size bone defects still may need further enhancement of the bone regeneration potential of Spongostan™. Therefore, scaffolds with optimised nano- and microtopography additionally loaded with stem cells or growth factors may even more efficiently achieve bone regeneration in the patient. 

## 5. Conclusions

In the present study, we compared the in vivo bone regeneration potential of the two clinical proven synthetic bone substitute materials NanoBone^®^ and Actifuse with Spongostan™, a clinically applied collagen sponge normally used as a haemostatic agent. NanoBone^®^ and Actifuse (based on hydroxyapatite and calcium phosphate) resulted in comparable volumes of newly formed bone after transplantation into rat critical-size calvarial defects. Notably, the largest amount of newly formed bone and highest increase in bone mineral density was found in the Spongostan™ group compared to NanoBone^®^ and Actifuse, suggesting the distinct nano- and microtopography of wet Spongostan™ to account for this superior regenerative capacity. 

## Figures and Tables

**Table 1:** Descriptive statistics for the newly formed bone volume (mm^3^).

New Bone Volume	Group A	Group B	Group C	Group D
Mean	2.97	6.69	6.03	7.98
Median	2.56	6.79	6.26	8.12
Standard deviation	1.31	1.70	1.03	1.68
Minimum	1.70	2.44	3.74	4.94
Maximum	6.17	8.75	7.37	1.83

**Table 2:** Bone mineral density (g/cm^3^)—descriptive statistics.

	Group A		Group B		Group C		Group D	
BMD	New	Intact	New	Intact	New	Intact	New	Intact
Mean	0.77	0.67	0.73	0.67	0.69	0.64	0.80	0.61
Median	0.78	0.68	0.73	0.67	0.69	0.62	0.80	0.61
Standard deviation	0.04	0.04	0.03	0.05	0.07	0.07	0.02	0.06
Minimum	0.71	0.60	0.68	0.60	0.61	0.57	0.78	0.51
Maximum	0.82	0.74	0.77	0.75	0.79	0.76	0.83	0.71

## Data Availability

The data presented in this study are available on request from the corresponding author.
